# Supplementary material for: Maternal mortality in Goiás: Causes of maternal death and barriers to underreporting
Source: PLOS Glob Public Health. 2025 Dec 3;5(12):e0004511. doi: 10.1371/journal.pgph.0004511 (PMC12674508; doi:10.1371/journal.pgph.0004511)
Supplement: S1 Table — (DOCX) [file pgph.0004511.s001.docx]

S1 Table. Codes used to select deaths of women of childbearing age, with presumption of maternal death, Brazil.

| **Description** | **ICD-10 Codes** |
| --- | --- |
| Septicemia | A400-A419 |
| Pelviperitonitis | A542 |
| Disseminated intravascular coagulation | D65 |
| Epilepsy | G400-G409 |
| Systemic arterial hypertension | I10 |
| Acute myocardial infarction | I210-I219 |
| Pulmonary embolism | I269 |
| Cardiomyopathy | I429 |
| Cardiac arrest | I469 |
| Congestive heart failure | I500 |
| Myocardial failure | I509 |
| Cerebrovascular accident | I64 |
| Acute intracranial hypertension | G932 |
| Thromboembolism | I740-I749 |
| Influenza | J100-J119 |
| Pneumonias | J120-J129, J13, J14, J150-J159, J160-J169, J180-J189 |
| Acute pulmonary edema | J81 |
| Peritonitis | K650-K659 |
| Acute hepatic failure | K720 |
| Acute renal failure | N170-N179 |
| Endometritis | N710-N719 |
| Pelviperitonitis | N733-N739 |
| Convulsive seizure | R568 |
| Hypovolemic shock | R571 |
| Hemotoxic shock | R578 |
| Hemorrhage | R58 |
| Unassisted death | R98 |
| Unknown cause | R99 |
| Anaphylactic shock | Y579 |
| Anesthetic shock | Y480-Y489 |

Brasil. Ministério da Saúde. Secretaria de Vigilância em Saúde. Coordenação Geral de Informação e Análise Epidemiológica. Protocolos de codificações especiais em mortalidade. Brasília: Ministério da Saúde; 2013
